# Gene expression profiling associated with the progression to poorly differentiated thyroid carcinomas

**DOI:** 10.1038/sj.bjc.6605340

**Published:** 2009-10-06

**Authors:** J M Pita, A Banito, B M Cavaco, V Leite

**Affiliations:** 1 Centro de Investigação de Patobiologia Molecular (CIPM), Instituto Português de Oncologia de Lisboa Francisco Gentil, Lisboa 1099-023, Portugal; 2 Centro de Estudos de Doenças Crónicas (CEDOC), Faculdade de Ciências Médicas da Universidade Nova de Lisboa, Lisboa 1169-056, Portugal; 3 Serviço de Endocrinologia, Instituto Português de Oncologia de Lisboa Francisco Gentil, Lisboa 1099-023, Portugal

**Keywords:** dedifferentiation, genome-wide expression, oligonucleotide microarray, poorly differentiated thyroid carcinoma, molecular signatures

## Abstract

**Background:**

Poorly differentiated thyroid carcinomas (PDTC) represent a heterogeneous, aggressive entity, presenting features that suggest a progression from well-differentiated carcinomas. To elucidate the mechanisms underlying such progression and identify novel therapeutic targets, we assessed the genome-wide expression in normal and tumour thyroid tissues.

**Methods:**

Microarray analyses of 24 thyroid carcinomas – 7 classic papillary, 8 follicular variants of papillary (fvPTC), 4 follicular (FTC) and 5 PDTC – were performed and correlated with *RAS*, *BRAF*, *RET/PTC* and *PAX8-PPARG* alterations. Selected genes were validated by quantitative RT–PCR in an independent set of 28 thyroid tumours.

**Results:**

Unsupervised analyses showed that gene expression similarity was higher between PDTC and fvPTC, particularly for tumours harbouring *RAS* mutations. Poorly differentiated thyroid carcinomas presented molecular signatures related to cell proliferation, poor prognosis, spindle assembly checkpoint and cell adhesion. Compared with normal tissues, PTC had 307 out of 494 (60%) genes over-expressed, FTC had 137 out of 171 (80%) genes under-expressed, whereas PDTC had 92 out of 107 (86%) genes under-expressed, suggesting that gene downregulation is involved in tumour dedifferentiation. Significant *UHRF1* and *ITIH5* deregulated gene expression in PDTC, relatively to normal tissues, was confirmed by quantitative RT–PCR.

**Conclusion:**

Our findings suggest that fvPTC are possible precursors of PDTC. Furthermore, *UHRF1* and *ITIH5* have a potential therapeutic/prognostic value for aggressive thyroid tumours.

Most thyroid neoplasias derive from follicular cells and show a wide range of biological behaviours from indolent to highly invasive cancers (DeLellis *et al*, 2004). Well-differentiated thyroid cancers (WDTC), such as papillary thyroid carcinoma (PTC) and follicular thyroid carcinoma (FTC), are usually treated successfully with surgery and radioactive iodine; however, poorly differentiated thyroid carcinoma (PDTC) and anaplastic (or undifferentiated) thyroid carcinoma (ATC) can behave aggressively with no effective form of treatment (Patel and Shaha, 2006).

Previous reports suggest a model of progression from WDTC to PDTC and to ATC. PDTC show limited follicular cell differentiation and are, both morphologically and behaviourally, positioned between well- and undifferentiated carcinomas (DeLellis *et al*, 2004). Indeed, cases of WDTC containing areas of poor- or undifferentiation, as well as, cases of PDTC/ATC containing well-differentiated areas, have been widely detected (Lam *et al*, 2000). Progression is further suggested by the sequential increase in chromosomal abnormalities from WDTC to PDTC and ATC (Wreesmann *et al*, 2002; Rodrigues *et al*, 2004). Mutations in the *RAS* and *BRAF* genes also support a model of tumour progression, as the frequency of these events in PDTC is midway between well-differentiated and undifferentiated carcinomas, rather than being randomly distributed (Garcia-Rostan *et al*, 2003; Nikiforova *et al*, 2003). Other alterations, such as tumour suppressor *TP53* mutations, are specifically found in PDTC and ATC, and are often associated with *RAS* or *BRAF* mutations (Quiros *et al*, 2005; Wang *et al*, 2007), suggesting an accumulation of events during progression. Nevertheless, it is not clear whether PDTC derive from either PTC or FTC, or whether they arise *de novo*. In addition, the genetic and epigenetic mechanisms underlying the process remain ill defined.

Genome-wide expression analysis has been successfully used to identify molecular signatures, improving the diagnosis and prognosis of several types of tumours (Quackenbush, 2006). For thyroid neoplasias, one of the earliest reports of genome-wide expression analysis described a consistent gene expression profile that distinguished PTC from normal cells (Huang *et al*, 2001). Most of the genes identified in this work were corroborated in subsequent studies. Gene expression studies have also been used to differentiate benign from malignant thyroid tumours, and correlate gene expression patterns with specific mutations or rearrangements in PTC and FTC (for review, see Eszlinger *et al*, 2007).

To our knowledge, only two studies have addressed the genome-wide expression of PDTC. One of these studies compared gene expression of PDTC and ATC cell lines to normal thyroid tissue, and showed that these cells presented largely altered expression profiles that have been associated with the cancer process (Rodrigues *et al*, 2007). Although the authors confirmed some of the abnormal expressed genes in primary tumours, it has been shown that immortal cell lines may not fully reflect the functional aspects of the tumours, and that some molecular processes might be specifically acquired during the immortalisation step (Dairkee *et al*, 2004). In the other study, which used WDTC, PDTC and ATC primary tumours, deregulation of different molecular pathways, such as the MAPK signalling pathway, focal adhesion and cell motility, cell proliferation and cell-cycle progression, was associated with dedifferentiation in PDTC and ATC (Montero-Conde *et al*, 2008).

In this study, we used the array platform *GeneChip Human Genome U133 Plus 2.0* (*HG-U133 Plus 2.0*) to analyse the expression of a wide range of genes (>30 000) in well- and poorly differentiated thyroid tumours and, to correlate, for the first time, gene expression with BRAF, RAS, RET/PTC and PAX8-PPARG alterations.

## Materials and methods

### Tissue samples

Both tumour and normal thyroid tissue samples were obtained at time of surgery, and were immediately frozen in liquid nitrogen. Histological classifications followed the criteria described in World Health Organization (WHO) classification of thyroid tumours (DeLellis *et al*, 2004). All samples were obtained with permission, and the project was approved by our institution ethical committee.

The microarray sample set consisted of a total of 24 tumour samples – 5 PDTC, 7 classic PTC (cPTC), 8 follicular variants of PTC (fvPTC) and 4 FTC (Supplementary Table 1). A pool of human thyroid total RNA obtained from 65 Caucasian individuals with 18–61-years old, whom died from sudden death (BD Bioscience, Franklin Lakes, NJ, USA), and 2 normal tissue samples taken from the opposite lobe of thyroid tumours, were also processed.

An independent sample set, consisting of five PDTC, seven cPTC, seven fvPTC, nine FTC and six normal thyroid tissues taken from the opposite lobe of thyroid tumours, was used for quantitative real-time RT–PCR. Expression was also studied in two poorly differentiated thyroid cancer cell lines, T243 and T351 (described earlier by Rodrigues *et al*, 2007), kindly supplied by Dr Lúcia Roque, from Centro de Investigação de Patobiologia Molecular (CIPM), Instituto Português de Oncologia de Lisboa Francisco Gentil, Lisbon, Portugal.

### Total RNA isolation/extraction

Total RNA was extracted and purified using the RNeasy Mini kit (Quiagen, Hamburg, GmbH, Germany) according to the manufacturer's protocol, and quantified by UV spectrophotometry (NanoDrop ND-1000, Thermo Fisher Scientific, Wilmington, DE, USA). RNA integrity was assessed by micro capillary electrophoresis (Agilent 2100 Bioanalyzer, Santa Clara, CA, USA) and samples with RNA Integrity Number equal or higher than 7.7 were selected for microarray analysis.

### RNA processing and hybridisation

RNA samples were processed following the one-cycle eukaryotic target labelling protocol from *Affymetrix*, and were hybridised using the *HG-U133 Plus 2.0 Array* (Affymetrix, Santa Clara, CA, USA). Hybridisation results were scanned using the *GeneChip Scanner 3000* and stored in the *GeneChip Operating Software*.

### Microarray data analysis

*Partek Genomics Suite Software* (Partek Inc, St Louis, MO, USA) was used for unsupervised analyses. First, array data were normalised and the expression levels were determined applying the robust multi-array average method (Irizarry *et al*, 2003), and data were corrected for non-biological factors. Samples were represented three dimensionally, according to the expression levels of all probe sets, by principal components analysis (PCA). Probe-sets data were also used to obtain a dendogram of the samples, by hierarchical clustering, with the Pearson correlation coefficient.

*DNA-ChipAnalyzer (dChip)* 2006 software (Li and Wong, 2001) was used to obtain differentially expressed genes between groups. Arrays were normalised with the invariant set normalisation method and expression levels were calculated by model-based expression analysis with perfect match-only model. Probe sets that were absent in all samples or those that did not change across samples (coefficient of variation lower than 0.2 and higher than 10) were eliminated from further analysis. Probe sets were considered to be differentially expressed between two groups when the lower 90% limit of the confidence interval of the fold change (ratio of the expression level in the two groups) was equal or higher than two-fold, with an unpaired *t*-test considered significant at *P*⩽0.01. Onto-Express from Onto-Tools package (Khatri *et al*, 2002) was used to classify genes differentially expressed according to their biological role.

Gene set enrichment analysis (GSEA) software (Subramanian *et al*, 2005) was used to determine whether members of defined groups of genes, which share common features (gene sets), are preferentially placed towards the top or the bottom of a list of genes. In this list, genes were ranked according to the differential expression between two sample groups. This method was applied using two catalogues of gene sets: one whose products are involved in specific pathways/functions and another defined by expression neighbourhoods, which indicates molecular signatures associated with cancer-related genes. Statistical significance was estimated by a nominal *P* value obtained by phenotype permutation. *P* values were corrected for multiple hypothesis testing using false discovery rate and family wise-error rate (FWER). Gene sets were considered significant at *P*<0.05 and with FWER⩽0.25.

### First-strand cDNA synthesis

cDNA was synthesised from 1 *μ*g of total RNA (for cDNA sequence analysis) or 2 *μ*g of total RNA (for quantitative RT–PCR), at 37°C for 90 min, using random primer p(dN)_6_ (Roche Diagnostics Corporation, Indianapolis, IN, USA) and SuperScript II reverse transcriptase (Invitrogen, Paisley, UK).

### Mutational analysis of the *RAS*, *BRAF* genes and *PAX8-PPARG*, *RET/PTC* rearrangements

Mutational analysis was undertaken using cDNA from the tumour samples of the microarray set. PTC were screened for *BRAF* mutations and rearrangements of *RET/PTC* and, in addition, follicular variants were also analysed for *RAS* mutations and *PAX8-PPARG* rearrangements. FTC were screened for *RAS* and *PAX8-PPARG* rearrangements. PDTC were analysed for *BRAF*, *RAS* and *PAX8-PPARG* genes. Primers were designed to amplify exon 15 of the *BRAF* gene, exons 2 and 3 of the *N*-, *KRAS* genes and exons 1 and 2 of the *HRAS* gene. Primers flanking the respective fusion points were used to screen the presence of *RET/PTC1*, *RET/PTC2*, *RET/PTC3* and *PAX8-PPARG* fusion transcripts, as described earlier (Marques *et al*, 2002; Rebelo *et al*, 2003). Sequencing analysis, to search for mutations and to confirm the rearrangements, was performed with the same primers as for PCR, using the Big Dye Terminator v1.1 Cycle Sequencing kit (Applied Biosystems, Foster City, CA, USA), according to the manufacturer's protocol. Sequencing products were separated in an ABI Prism 310 Genetic Analyser (Applied Biosystems) and analysed with the Sequence Analysis Software version 3.4.1 (Applied Biosystems). Primer sequences and assay conditions are available on request.

### Quantitative real-time RT–PCR

Real-time RT–PCR assays were performed in 96-well reaction plates (MicroAmp Optical 96-Well Reaction Plate; Applied Biosystems) on an ABI Prism 7900 HT Sequence Detection System (Applied Biosystems) with the SDS Software version 2.3 (Applied Biosystems). PCR amplifications were performed using for each gene, pre-developed primers and probe (Inventoried TaqMan Gene Expression Assays ID: Hs00218544_m1 (*PBK*); Hs00273589_m1 (*UHRF1*); Hs00228960_m1 (*ITIH5*); Applied Biosystems), and TaqMan Universal PCR Master Mix (Applied Biosystems), according to the manufacturer's protocol. To normalise differences in the amount of template used, glyceraldehyde-3-phosphate dehydrogenase (*GAPDH*) transcript was used as an endogenous control (Pre-Developed TaqMan Assay Reagents Human *GAPDH*; Applied Biosystems). Two-fold serial dilutions were used to apply the relative standard curve method. A pool of five normal thyroid tissues was used as calibrator to determine the relative expression in samples. All reactions, including a control without template, were performed in triplicate.

Quantitative RT–PCR results were analysed using the GraphPad Prism version 4.00 (GraphPad Software, Inc). Intensity levels were calculated as mean±standard error of the mean (s.e.m.). Comparisons between sample groups were performed using the Kruskal–Wallis with Dunn's Multiple Comparison test, because samples distribution was not Gaussian or variances between groups were not equal. Correlations of quantitative RT–PCR data with other variables were performed using the Pearson or the Spearman correlations (for a non-Gaussian distribution). The correlations and differences between group means were considered significant at *P*<0.05.

## Results

### Mutation screening

Tumour samples were screened for mutations in MAPK pathway effectors, which are frequently mutated in thyroid cancer (Supplementary Table 1). The *BRAF* V600E substitution was only present in cPTC, accounting for 57.1% (four out of seven) of the cases. On the other hand, mutations of *N*- or *KRAS* genes were observed in 50% (four out of eight) of the fvPTC and in 40% (two out of five) of PDTC. All FTC (*n*=4) were *RAS* negative. The *PAX8-PPARG* fusion gene was found in 12.5% (one out of eight) of fvPTC and in 25% (one out of four) of FTC. No *RET/PTC1*, -*2* or -*3* rearrangements were identified in PTC. However, as other rearrangements involving the *RET* (Ciampi *et al*, 2007) or *NTRK1* (Pierotti and Greco, 2006) genes have also been described in PTC, we specifically analysed *RET* and *NTRK1* microarray mRNA expression in PTC negative for mutations. In one cPTC (sample 2 – Supplementary Table 1), a 20-fold increase in *RET* expression was detected in comparison to the other samples. FISH confirmed the presence of a *RET/PTC* rearrangement in 37% (71 out of 194) of these tumour cells (data not shown).

### Unsupervised analyses for global gene expression profiling

**Figure 1:**
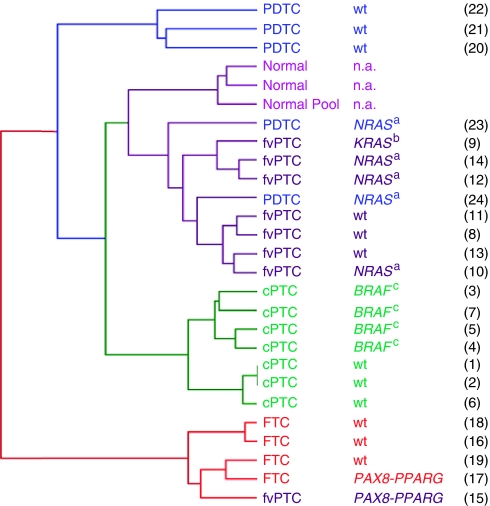
Global gene expression similarity between samples using the unsupervised hierarchical clustering method. In the dendogram, distance separating samples represents the gene expression resemblance between them. The result of mutational analysis for each tumour specimen is shown. Wild-type (wt) label denotes absence of mutation in screened genes; ^a^Q61R mutant of *NRAS*; ^b^G13R mutant of *KRAS*; ^c^V600E mutant of *BRAF*; in parenthesis is indicated the sample number assigned in Supplementary Table 1. cPTC=classic papillary thyroid carcinoma; FTC=follicular thyroid carcinoma; fvPTC=follicular variant of papillary thyroid carcinoma; PDTC=poorly differentiated thyroid carcinoma; n.a.=not applicable.

We carried out unsupervised analyses to examine the relationship between gene expression, tumour histotype and mutational status. Global gene expression similarity between the 27 samples was examined using hierarchical clustering (Figure 1). As represented on the dendrogram, distinct profiles separated FTC from the other tumours. Interestingly, a case of fvPTC diffuse (or multinodular) with a *PAX8-PPARG* rearrangement clustered with FTC. Different molecular signatures were present in the PTC sub-set: cPTC formed a separate sub-group from fvPTC and, among these PTC subtypes, samples with *RAS*/*BRAF* mutations were separated from samples without mutations. By PCA of data for all probe sets, which represents the samples three dimensionally according to the global gene expression profile, the FTC and the fvPTC diffuse (or multinodular) also formed a group apart from the other tumours and normal thyroid samples, which tended to cluster together (Supplementary Figure 1; Supplementary Movie 1). PDTC samples, particularly those with *RAS* mutations, clustered with PTC in both representations.

### Genes differentially expressed between tumours and normal tissue

**Figure 2:**
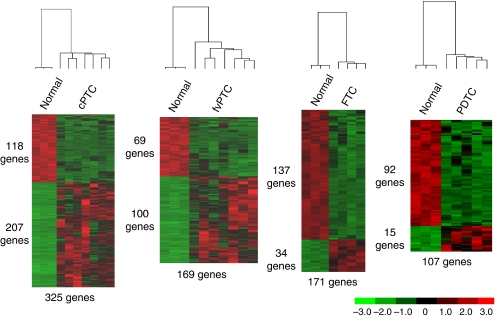
Expression profile of the genes differentially expressed between tumours and normal tissues. Each tumour histology was compared with the normal thyroid tissues and expression levels of the genes differentially expressed were represented. Expression levels are indicated by colour intensities in which green and red correspond, respectively, to a lower and a higher expression than the mean value for the gene, in all samples being compared. On the left of each profile, the number of under- and over-expressed genes in the tumour set is shown. At the bottom, the total number of differentially expressed genes is indicated. Only one probe set was considered for each gene. cPTC=classic papillary thyroid carcinoma; FTC=follicular thyroid carcinoma; fvPTC=follicular variant of papillary thyroid carcinoma; PDTC=poorly differentiated thyroid carcinoma.

Differentially expressed genes were defined as those with an expression level equal, or higher, than two-fold in a group relatively to another, with a *P* value⩽0.01. We compared each tumour group (cPTC, fvPTC, FTC and PDTC) with the three normal thyroid samples, and we found over-expression of about 60% of probe sets for both cPTC and fvPTC, whereas in FTC and PDTC, about 80% of the probe sets were under-expressed (Figure 2). PDTC had 92 downregulated genes relatively to normal tissues (Supplementary Table 2), but only 15 out of the 107 genes differentially expressed were over-expressed (Table 1).

The biological processes mainly represented by the probe sets differentially expressed between thyroid tumours and normal tissues were the signal transduction, cell adhesion, regulation of transcription and cell proliferation/cell cycle (Supplementary Figure 2). We were also able to identify 11 probe sets that were under-expressed in all tumours comparatively to normal tissues (Table 2).

### Genes specific for PDTC

The PDTC group had 3, 8 and 1 over-expressed probe sets and 11, 154 and 59 under-expressed probe sets compared with FTC, cPTC and fvPTC, respectively (data not shown). Only two probe sets were consistently under-expressed in the PDTC tumour set comparatively to the WDTC (Table 1).

### GSEA for PDTC

GSEA is another method for interpreting gene expression data that focus on groups of genes sharing common biological function, chromosomal location or regulation. This approach can show important effects on pathways, which might be missed in single-gene analysis (Subramanian *et al*, 2005). We applied this methodology to identify pathways altered in thyroid tumour progression. There were no statistically significant functional-defined gene sets enriched in PDTC samples *vs* WDTC, still we analysed the 20 most relevant results (Table 3). The budding uninhibited by benzimidazoles 1 homolog (yeast) (*BUB1*) was the most represented gene being present in 13 of the 20 gene sets. Cell division cycle 2, G1–S and G2–M (*CDC2*) and Cyclin B2 (*CCNB2*) were represented in 12 of the 20 gene sets, and 10 gene sets contained MAD2 mitotic arrest-deficient-like 1 (yeast) (*MAD2L1*), topoisomerase (DNA) II *α* 170 kDa (*TOP2A*), cyclin-dependent kinase inhibitor 3 (CDK2-associated dual specificity phosphatase) (*CDKN3*) and centromere protein A (*CENPA*). In addition, statistically significant molecular signatures of four deregulated genes, which are involved in the cancer process, were identified in PDTC (Table 3 – Expression neighbourhoods-defined gene sets).

### Validation of microarray gene expression

**Figure 3:**
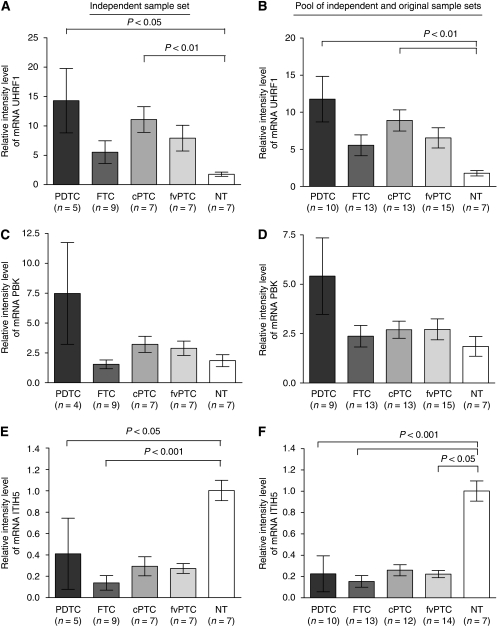
Expression of the *UHRF1*, *PBK* and *ITIH5* genes in different tumour histotypes, assessed by quantitative RT–PCR. Relative mRNA levels for *UHRF1* (**A**, **B**), *PBK* (**C**, **D**) and *ITIH5* (**E**, **F**) were assessed in an independent sample set (left panel) and in the entire sample set, comprising the microarray and the independent sample sets (right panel). Expression levels were normalised with the GAPDH expression and determined relatively to a calibrator. Error bars denote±s.e.m. The *P* values for difference in mean expression between groups were performed using the Kruskal–Wallis with Dunn's Multiple Comparison test. These genes could not be assayed in one cPTC from the microarray set, *PBK* could not be evaluated in one PDTC sample, as well as, *ITIH5* in one fvPTC and one cPTC from microarray set. cPTC=classic papillary thyroid carcinoma; FTC=follicular thyroid carcinoma; fvPTC=follicular variant of papillary thyroid carcinoma; PDTC=poorly differentiated thyroid carcinoma; NT=normal thyroid tissue.

Real-Time RT–PCR was performed to validate three genes differentially expressed between PDTC and normal thyroid samples: ubiquitin-like, containing PHD and RING finger domains, 1 (*UHRF1*), PDZ-binding kinase (*PBK*) and inter-alpha (globulin) inhibitor H5 (*ITIH5*). This validation was processed in an independent sample set of 28 tumours (Figure 3). *UHRF1* and *PBK* had increased expression in all tumour samples relatively to normal tissue samples, but the highest expression levels were detected in PDTC. Differences in the *UHRF1* expression (Figure 3B) between PDTC and normal tissue were statistically significant (11.77±3.07 *vs* 1.77±0.36; *P*<0.01), even if only considering the expression in the independent set (14.30±5.49 *vs* 1.77±0.36; *P*<0.05) (Figure 3A). On the other hand, differences in the PBK expression were not statistically significant (Figures 3C and D). *ITIH5* expression was decreased in all tumours samples relatively to normal tissues (Figures 3E and F). Statistically significant differences were detected in all tumour groups relatively to normal samples, except in cPTC (PDTC: 0.22±0.17, *P*<0.001; FTC: 0.15±0.05, *P*<0.001; fvPTC: 0.22±0.03, *P*<0.05 *vs* normal: 1.00±0.10). Differences were also significant when considering the independent set of samples (PDTC: 0.41±0.33, *P*<0.05; FTC: 0.14±0.07, *P*<0.001 *vs* normal: 1.00±0.10). Additionally, we assessed *UHRF1* and *ITIH5* expressions in two PDTC cell lines. No significant increase in *UHRF1* expression was detected (mean±standard deviation: 1.86±0.16 and 1.43±0.16 *vs* 1.77±0.36). On the other hand, *ITIH5* expression was undetectable (Ct values>37) in both cell lines. Correlation of quantitative RT–PCR data with the expression levels obtained in the microarray analysis was statistically significant (Pearson correlation *r*=0.61 for *UHRF1* with *P*=0.0015; Spearman correlation *r*=0.70 for *PBK* with *P*=0.0001; Pearson correlation *r*=0.94 for *ITIH5* with *P*<0.0001). There was no correlation of gene expression with tumour size, gender or age, except for *ITIH5*, whose decreased expression was associated with larger tumours (Spearman correlation *r*= −0.33 with *P*=0.02).

## Discussion

The more aggressive thyroid carcinomas (PDTC and ATC) have high malignant potential and it is not yet clear whether they arise from pre-existing indolent WDTC or whether they arise *de novo*. Some PDTC cases bear areas of pre-existing PTC and have a significant prevalence of *BRAF* mutations (Nikiforova *et al*, 2003). Others, instead of *BRAF*, frequently display *RAS* mutations (Garcia-Rostan *et al*, 2003), which are typically detected in follicular thyroid adenomas, FTC and fvPTC. Comparative Genomic Hybridisation (CGH) studies showed that among 11 copy number changes present in PTC, 8 were also present in the PDTC set, thus suggesting common genetic pathways (Wreesmann *et al*, 2002).

In our work, we were able to identify distinct gene expression profiles among different thyroid tumour histotypes. Our results suggest that PDTC have a gene expression profile closer to PTC, in particular to the follicular variant, than to FTC. In fact, for PDTC harbouring *RAS* mutations, a clear similarity to the gene profile of *RAS*-mutated fvPTC was observed. Interestingly, these *RAS*-mutated PDTC, presented papillary like nuclei. In keeping with our findings, it has been observed that fvPTC, in contrast to cPTC, are more frequently aneuploid (Wreesmann *et al*, 2004), a feature common in PDTC. Therefore, fvPTC are likely to be precursors of PDTC, particularly those cases harbouring *RAS* mutations.

We also analysed the differential gene expression between tumours and normal thyroid tissues. PTC cases had slightly more over- than under-expressed probe sets, confirming previous reports (Huang *et al*, 2001). On the other hand, FTC and PDTC had clearly a predominance of downregulated probe sets, which is also in accordance with others (Aldred *et al*, 2003; Rodrigues *et al*, 2007). Studies have shown that allelic losses are clearly more frequent in FTC and PDTC than in PTC (Ward *et al*, 1998; Rodrigues *et al*, 2004). This could account for the differences in gene expression as a genomic loss could, theoretically, originate under-expression of genes. Epigenetic mechanisms, such as DNA hypermethylation, are also likely to explain these expression profiles. Indeed, increased frequency of hypermethylated CpG islands is a common alteration in tumour progression.

Eleven probe sets were simultaneously under-expressed in all tumours relatively to normal tissues, suggesting that these genes may have important suppressor activity in thyroid tumourigenesis. Among these, metallophosphoesterase domain containing 2 (*MPPED2*) and cellular retinoic acid-binding protein 1 (*CRABP1*) under-expression have already been observed in thyroid tumours (Griffith *et al*, 2006).

As observed earlier in other genome-wide studies, comparing clinically aggressive PTC with differentiated PTC cases (Fluge *et al*, 2006) or comparing PDTC with normal tissue (Rodrigues *et al*, 2007) or with WDTC (Montero-Conde *et al*, 2008), we found that many of the genes differentially upregulated in PDTC relatively to normal tissues were associated with the cell-cycle, indicating that the deregulation of this process is crucial in the progression to more aggressive thyroid tumours. In particular, we identified genes with major roles in mitosis, such as CDC28 protein kinase regulatory subunit 2 (*CKS2*) and cyclin E2 (*CCNE2*), which have been reported as over-expressed in various types of tumours (Gudas *et al*, 1999; Scrideli *et al*, 2008).

We selected three genes for real-time RT–PCR analysis, which were shown in the microarray analysis, to be differentially over-expressed (*PBK* and *UHRF1*) and under-expressed (*ITIH5*) between PDTC and normal thyroid tissues. *UHRF1* and *ITIH5* expressions were statistically different between PDTC and normal thyroid samples. Although not statistically significant, *PBK* had higher expression levels in PDTC compared with normal thyroid. Therefore, *PBK* is also a potential therapeutic target, as it encodes a mitotic protein, member of MAPK kinases family, which was found to be over-expressed in haematological (Nandi *et al*, 2004) and breast tumours (Park *et al*, 2006), and in PDTC cell lines (Rodrigues *et al*, 2007). *UHRF1* over-expression has been already reported in lung (Jenkins *et al*, 2005) and breast (Hopfner *et al*, 2000) cancers. By conventional CGH analyses, the chromosomal *locus* 19p13.3, where *UHRF1* is located, was identified as a common region of chromosomal gains in Hürthle cell thyroid neoplasms (Wada *et al*, 2002) and recently, using array-CGH, gains involving the 19p13 region were also found in 67% of ATC (Lee *et al*, 2008). *UHRF1* encodes a nuclear protein that transcriptionally regulates TOP2A (Hopfner *et al*, 2000), an enzyme that catalyses the breaking and rejoining of DNA strands, during transcription. Interestingly, *TOP2A* showed a five-fold increase in PDTC *vs* normal tissues (*P*=0.02). In addition, UHRF1 is known to regulate the retinoblastoma protein (Jeanblanc *et al*, 2005) and is involved in the DNA damage response (Jenkins *et al*, 2005). More recently, an essential role for UHRF1 in the control of DNA methyltransferase 1 (DNMT1), the protein responsible for DNA methylation maintenance in mammalian cells, has also been reported (Bostick *et al*, 2007; Sharif *et al*, 2007). UHRF1 and DNMT1 interactions have been shown to be involved in VEGF regulation, a major pro-angiogenic protein (Achour *et al*, 2008).

Classification according to biological functions showed that about 8.5% of the genes differentially expressed between PDTC and normal tissues were downregulated and all related to cell adhesion. Interestingly, by further analysis, we found that under-expressed genes were mainly related to the cell membrane, encoding receptors, transmembranar or extracellular proteins. In agreement with this, the *ITIH5* gene, which may have an essential function in cell attachment and invasion, was under-expressed in PDTC tumours. *ITIH5* is a recently discovered member of the inter-alpha (globulin) inhibitor heavy chains (*ITIH*) gene family (Himmelfarb *et al*, 2004). In particular, the main function of ITIH is based on their covalent linkage to hyaluronic acid, the major component of the extracellular matrix. Therefore, deregulation of ITIH proteins affects the stability of the extracellular matrix and so, may promote tumour invasion and metastasis (Bost *et al*, 1998). Accordingly, *ITIH* genes have been shown to be downregulated in a variety of human tumours and have been proposed as tumour suppressor or metastasis repressor genes (Hamm *et al*, 2008). *ITIH5* downregulation in breast cancer, caused by promoter hypermethylation, is associated with poorer clinical outcome, and reduced protein expression was proved to be a bad prognostic marker in invasive node-negative patients (Veeck *et al*, 2008). In fact, we found that lower expression of *ITIH5* was statistically associated with larger tumours, as well as with more aggressive cases, such as PDTC (reaching undetectable levels in the two PDTC cell lines). Interestingly, we could also observe that extensively invasive FTC had lower *ITIH5* expression levels than minimally invasive ones.

Compared with WDTC, PDTC had enriched gene sets (represented by over-expressed genes) associated earlier with cell cycle and poor prognosis signatures. Interestingly, one of these sets corresponded to a meta-signature of genes differentially over-expressed in undifferentiated relatively to well-differentiated cancers of different tissues (Rhodes *et al*, 2004). Among the most represented genes in these sets were cell-cycle regulators (*CDC2*, *CCNB2*, *CDKN3* and *TOP2A*), as well as genes with a role in the structure of the kinetochore and in the mitotic spindle assembly checkpoint (MSAC) (*CENPA*, *BUB1* and *MAD2L1*). We also found statistically significant molecular signatures associated with the *BUB1* and *BUB1 β* (yeast) (*BUB1B*) genes. Some of these genes were reported earlier to be over-expressed in advanced cases of thyroid tumours (Montero-Conde *et al*, 2008; Wada *et al*, 2008). These observations indicate that PDTC may have abnormalities in MSAC or in the attachment of kinetochores, which may compromise mitotic fidelity and contribute to chromosomal instability. Accordingly, we observed earlier (Banito *et al*, 2007) that four out of the five PDTC analysed in this study were aneuploid.

In the analysis of genes differentially expressed between PDTC and WDTC, we only identified two genes. One of these, the 3-phosphoinositide-dependent protein kinase-1 gene (*PDPK1*), encodes a protein responsible for protein kinase B or AKT activation. PI3K/AKT pathway has a central role in regulation of apoptosis, proliferation and cell-cycle progression and its abnormal activation is frequently found in cancers, including thyroid tumours (Shinohara *et al*, 2007). Unexpectedly, and contrary to other cancer types, we observed that *PDPK1* gene was under-expressed in PDTC.

The identification of molecular mechanisms involved in tumour progression is important in the design of new strategies for treating aggressive neoplasias, such as PDTC and ATC. For instance, over-expression of UHRF1 in PDTC samples, a protein that seems to be essential for DNMT1 function, indicates that UHRF1 targeting may offer a new therapeutic approach for PDTC cases. On the other hand, *ITIH5* downregulation may be an essential mechanism in thyroid tumourigenesis, especially in tumour metastasis. In addition, and similarly to breast cancer, ITIH5 may prove to be a useful prognostic marker.

## Figures and Tables

**Table 1:** Main characteristics of differentially expressed genes in poorly differentiated tumours

***Probe set***	**Gene name in array *HG-U133 Plus 2.0***	**Official symbol** a	**Accession number**	**Biological process** b	**LBFC**	***P*-value**
*Genes over-expressed in PDTC vs normal thyroid tissues*						
204170_s_at	CDC28 protein kinase regulatory subunit 2	*CKS2*	NM_001827	Cell cycle	5.43	4.68E–03
219148_at	PDZ binding kinase	*PBK*	NM_018492	Cell cycle	4.29	8.04E−03
202503_s_at	KIAA0101	*KIAA0101*	NM_014736	Cell cycle	3.46	5.80E−03
205034_at	Cyclin E2	*CCNE2*	NM_004702	Cell cycle	2.95	6.21E−03
202975_s_at	Rho-related BTB domain containing 3	*RHOBTB3*	N21138	—	2.94	9.42E−03
218096_at	1-acylglycerol-3-phosphate *O*-acyltransferase 5 (lysophosphatidic acid acyltransferase, epsilon)	*AGPAT5*	NM_018361	Phospholipid metabolism	2.76	4.23E−03
225655_at	Ubiquitin-like, containing PHD and RING finger domains, 1	*UHRF1*	AK025578	Cell cycle	2.61	8.96E−03
229551_x_at	Zinc-finger protein 367	*ZNF367*	N62196	Transcription regulation	2.59	6.36E−04
220608_s_at	Homo sapiens PRO1914 protein (PRO1914)	*ZNF770*	NM_014106	Transcription regulation	2.58	2.73E−03
222848_at	Leucine zipper protein FKSG14	*CENPK*	BC005400	Transcription regulation	2.53	4.85E−04
224726_at	Mindbomb homolog 1 (Drosophila)	*MIB1*	W80418	*Notch* signaling	2.38	1.98E−04
220145_at	ASAP	*MAP9*	NM_024826	Cell cycle	2.07	6.36E−03
218819_at	DEAD/H (Asp-Glu-Ala-Asp/His) box polypeptide 26	*INTS6*	NM_012141	snRNA processing	2.05	3.30E−03
235609_at	BRCA1 interacting protein C-terminal helicase 1	*BRIP1*	BF056791	DNA DBS repair	2.05	7.46E−03
203007_x_at	Lysophospholipase I	*LYPLA1*	AF077198	Phospholipid metabolism	2.03	2.24E−03
						
*Genes under-expressed in PDTC vs WDTC*						
225119_at	Chromatin modifying protein 4B	*CHMP4B*	AW299290	Protein transport	−2.97	0.00
204524_at	3-phosphoinositide-dependent protein kinase-1	*PDPK1*	NM_002613	Cell adhesion	−2.61	0.00

Abbreviations: DBS=double-strand breaks; LBFC=lower bound of fold change; snRNA=small nuclear RNA.

a Assigned in *EntrezGene*.

b Information taken from Online Mendelian Inheritance in Man (OMIM) or from *EntrezGene*. *P* values for difference in mean expression between groups were calculated using an unpaired *t*-test.

**Table 2:** Main characteristics of differentially expressed genes in the four thyroid tumour histotypes *vs* normal thyroid tissues

***Probe set***	**Gene name in array *HG-U133 Plus 2.0***	**Official symbol** a	**Accession number**	**Biological process** b	**LBFC**	***P*-value**
*Under-expressed genes in the four types of tumours vs normal thyroid tissues*						
205382_s_at	D component of complement (adipsin)	*CFD*	NM_001928	Immune response	−9.31	4.74E−03
204606_at	Chemokine (C-C motif) ligand 21	*CCL21*	NM_002989	Inflammatory response	−7.57	5.72E−03
235849_at	Hypothetical protein MGC45780	*SCARA5*	BE787752	Immune response	−6.38	9.25E−03
205350_at	Cellular retinoic acid binding protein 1	*CRABP1*	NM_004378	Retinoic acid metabolism	−6.13	5.13E−03
203060_s_at	3′-phosphoadenosine 5′-phosphosulfate synthase 2	*PAPSS2*	AF074331	Sulfur metabolism	−5.98	8.55E−04
212713_at	Microfibrillar-associated protein 4	*MFAP4*	R72286	Cell adhesion	−4.20	3.81E−04
1556427_s_at	Similar to hypothetical protein	*LOC221091*	AL834319	—	−4.07	5.57E−03
219778_at	Zinc-finger protein, multitype 2	*ZFPM2*	NM_012082	Transcription regulation	−4.03	7.60E−03
205413_at	Chromosome 11 open reading frame 8	*MPPED2*	NM_001584	Nervous system development	−3.59	9.00E−05
206201_s_at	Mesenchyme homeo box 2 (growth arrest-specific homeo box)	*MEOX2*	NM_005924	Development	−2.92	5.85E−03
217525_at	Olfactomedin-like 1	*OLFML1*	AW305097	Cell proliferation	−2.64	2.34E−04

Abbreviation: LBFC=lower bound of fold change.

a Assigned in *EntrezGene.*

b Information taken from Online Mendelian Inheritance in Man (OMIM) or from *EntrezGene*. *P* values for difference in mean expression between tumours and normal tissues were calculated using an unpaired *t*-test.

**Table 3:** Gene sets enriched in the poorly differentiated *vs* the well-differentiated groups

**Gene set name^a^**	**Gene set description**	**Nominal *P*-value**	**FDR**	**FWER**	**Reference**
**Functional-defined gene sets**					
LEE_TCELLS3_UP	Enriched in both intrathymic T progenitor cells and CD3^int^CD4^+^CD8^+^ thymocytes	1.01E−02	0.95	0.43	Lee *et al* (2004)
YU_CMYC_UP	C-Myc activated genes	6.93E−03	0.56	0.47	Yu *et al* (2005)
GREENBAUM_E2A_UP	Upregulated in E2A-deficient pre-B-cell lines	5.14E−03	0.44	0.51	Greenbaum *et al* (2004)
VANTVEER_BREAST_OUTCOME_ GOOD_VS_POOR_DN	Poor prognosis marker genes in breast cancer	7.61E−03	0.38	0.55	van’t Veer *et al* (2002)
MANALO_HYPOXIA_DN	Genes downregulated in human pulmonary endothelial cells under hypoxic conditions	8.89E−03	0.31	0.55	Manalo *et al* (2005)
ADIP_DIFF_CLUSTER5	Strongly upregulated at 24 h during differentiation of 3T3-L1 fibroblasts into adipocytes	2.40E−02	0.32	0.62	Burton *et al* (2002)
CANCER_UNDIFFERENTIATED_ META_UP	Genes commonly upregulated in undifferentiated cancer relative to well-differentiated cancer	1.64E−02	0.30	0.64	Rhodes *et al* (2004)
HUMAN_TISSUE_TESTIS	Genes expressed specifically in human testis tissue	1.15E−02	0.34	0.70	Su *et al* (2002)
ZHAN_MM_CD138_PR_VS_REST	Top ranked over-expressed genes in proliferation subgroup of bone marrow plasma cells from multiple myeloma patients	9.83E−03	0.31	0.70	Zhan *et al* (2006)
CROONQUIST_IL6_STARVE_UP	Genes upregulated in multiple myeloma cells exposed to cytokine IL6 *vs* IL6-starved cells	7.21E−03	0.29	0.71	Croonquist *et al* (2003)
BRCA_PROGNOSIS_NEG	Negatively correlated with metastasis and poor prognosis in breast cancer	1.37E−02	0.28	0.73	Van’t Veer *et al* (2002)
P21_ANY_DN	Downregulated after ectopic expression of p21 (*CDKN1A*) in ovarian cancer cell line	2.12E−02	0.27	0.74	Wu *et al* (2002)
DOX_RESIST_GASTRIC_UP	Upregulated in gastric cancer cell lines resistant to doxorubicin, compared with parent chemosensitive lines	1.94E−02	0.31	0.80	Kang *et al* (2004)
CROONQUIST_IL6_RAS_DN	Genes downregulated in multiple myeloma cells exposed to IL6 *vs* *NRAS* activating mutations cells	1.73E−02	0.29	0.80	Croonquist *et al* (2003)
REN_E2F1_TARGETS	E2F1 targets in primary fibroblast WI-38	2.84E−02	0.28	0.81	Ren *et al* (2002)
BREAST_DUCTAL_CARCINOMA_ GENES	Genes upregulated in high tumour grade breast tumours progressing from pre-invasive ductal carcinoma *in situ* to invasive ductal carcinoma	2.43E−02	0.30	0.84	a
SERUM_FIBROBLAST_CELLCYCLE	Cell-cycle-dependent genes, regulated after exposure to serum in a variety of human fibroblast cell lines	5.04E−02	0.28	0.85	Chang *et al* (2004)
BRENTANI_CELL_CYCLE	Cancer-related genes involved in the cell cycle	1.87E−02	0.28	0.86	Brentani *et al* (2003)
SHEPARD_CRASH_AND_BURN_ MUT_VS_WT_DN	Genes upregulated in wild-type zebrafish compared with the B-Myb loss-of-fuction mutants	2.13E−02	0.28	0.86	Shepard *et al* (2005)
GOLDRATH_CELLCYCLE	Cell-cycle genes induced during antigen activation of CD8^+^ T cells	2.50E−02	0.27	0.87	Goldrath *et al* (2004)
					
**Expression neighbourhoods-defined gene sets**					
GNF2_CKS1B	Expression neighbourhood of *CKS1B* in the GNF2 expression compendium	0.00	0.04	0.02	a
MORF_BUB1	Expression neighbourhood of *BUB1* in the MORF expression compendium	4.74E-03	0.14	0.10	a
MORF_BUB1B	Expression neighbourhood of *BUB1B* in the MORF expression compendium	0.00	0.20	0.19	a
GNF2_ESPL1	Expression neighbourhood of *ESPL1* in the GNF2 expression compendium	9.59E-03	0.18	0.21	a

Abbreviations: BUB1=budding uninhibited by benzimidazoles 1 homolog (yeast); BUB1B=budding uninhibited by benzimidazoles 1 homolog beta (yeast); CD=cluster of differentiation; IL6=interleukin 6; CDKN1A=cyclin-dependent kinase inhibitor 1A (p21, Cip1); CKS1B=CDC28 protein kinase regulatory subunit 1B; ESPL1=extra spindle pole bodies homolog 1 (*S. cerevisiae*); FDR=false discovery rate; FWER=family wise-error rate.

a Assigned in Molecular Signatures Database (www.broad.mit.edu/gsea/msigdb/index.jsp). References are supplied as supplementary data.
