# Supplementary material for: Gene expression profiling associated with the progression to poorly differentiated thyroid carcinomas
Source: Br J Cancer. 2009 Oct 6;101(10):1782–91. doi: 10.1038/sj.bjc.6605340 (PMC2778548; doi:10.1038/sj.bjc.6605340)
Supplement: Supplementary Information [file 6605340x2.doc]

**Supplementary Information**

**Supplementary References**

Brentani H, Caballero OL, Camargo AA, da Silva AM, da Silva WA Jr, Dias Neto E, Grivet M, Gruber A, Guimaraes PE, Hide W, Iseli C, Jongeneel CV, Kelso J, Nagai MA, Ojopi EP, Osorio EC, Reis EM, Riggins GJ, Simpson AJ, de Souza S, Stevenson BJ, Strausberg RL, Tajara EH, Verjovski-Almeida S, Acencio ML, Bengtson MH, Bettoni F, Bodmer WF, Briones MR, Camargo LP, Cavenee W, Cerutti JM, Coelho Andrade LE, Costa dos Santos PC, Ramos Costa MC, da Silva IT, Estécio MR, Sa Ferreira K, Furnari FB, Faria M Jr, Galante PA, Guimaraes GS, Holanda AJ, Kimura ET, Leerkes MR, Lu X, Maciel RM, Martins EA, Massirer KB, Melo AS, Mestriner CA, Miracca EC, Miranda LL, Nobrega FG, Oliveira PS, Paquola AC, Pandolfi JR, Campos Pardini MI, Passetti F, Quackenbush J, Schnabel B, Sogayar MC, Souza JE, Valentini SR, Zaiats AC, Amaral EJ, Arnaldi LA, de Araújo AG, de Bessa SA, Bicknell DC, Ribeiro de Camaro ME, Carraro DM, Carrer H, Carvalho AF, Colin C, Costa F, Curcio C, Guerreiro da Silva ID, Pereira da Silva N, Dellamano M, El-Dorry H, Espreafico EM, Scattone Ferreira AJ, Ayres Ferreira C, Fortes MA, Gama AH, Giannella-Neto D, Giannella ML, Giorgi RR, Goldman GH, Goldman MH, Hackel C, Ho PL, Kimura EM, Kowalski LP, Krieger JE, Leite LC, Lopes A, Luna AM, Mackay A, Mari SK, Marques AA, Martins WK, Montagnini A, Mourão Neto M, Nascimento AL, Neville AM, Nobrega MP, O'Hare MJ, Otsuka AY, Ruas de Melo AI, Paco-Larson ML, Guimarães Pereira G, Pereira da Silva N, Pesquero JB, Pessoa JG, Rahal P, Rainho CA, Rodrigues V, Rogatto SR, Romano CM, Romeiro JG, Rossi BM, Rusticci M, Guerra de Sá R, Sant' Anna SC, Sarmazo ML, Silva TC, Soares FA, Sonati Mde F, de Freitas Sousa J, Queiroz D, Valente V, Vettore AL, Villanova FE, Zago MA, Zalcberg H; Human Cancer Genome Project/Cancer Genome Anatomy Project Annotation Consortium; Human Cancer Genome Project Sequencing Consortium (2003) The generation and utilization of a cancer-oriented representation of the human transcriptome by using expressed sequence tags. *Proc Natl Acad Sci USA* **100**: 13418-13423

Burton GR, Guan Y, Nagarajan R, McGehee RE Jr (2002) Microarray analysis of gene expression during early adipocyte differentiation. *Gene* **293**: 21-31

Chang HY, Sneddon JB, Alizadeh AA, Sood R, West RB, Montgomery K, Chi JT, van de Rijn M, Botstein D, Brown PO (2004) Gene expression signature of fibroblast serum response predicts human cancer progression: similarities between tumors and wounds. *PLoS Biol* **2**: E7. doi:10.1371/journal.pbio.0020007

Croonquist PA, Linden MA, Zhao F, Van Ness BG (2003) Gene profiling of a myeloma cell line reveals similarities and unique signatures among IL-6 response, N-ras-activating mutations, and coculture with bone marrow stromal cells. *Blood* **102**: 2581-2592

Goldrath AW, Luckey CJ, Park R, Benoist C, Mathis D (2004) The molecular program induced in T cells undergoing homeostatic proliferation. *Proc Natl Acad Sci USA* **101**: 16885-16890

Greenbaum S, Lazorchak AS, Zhuang Y (2004) Differential functions for the transcription factor E2A in positive and negative gene regulation in pre-B lymphocytes. *J Biol Chem* **279**: 45028-45035. Erratum in: *J Biol Chem* (2005) **280**:16528

Kang HC, Kim IJ, Park JH, Shin Y, Ku JL, Jung MS, Yoo BC, Kim HK, Park JG (2004) Identification of genes with differential expression in acquired drug-resistant gastric cancer cells using high-density oligonucleotide microarrays. *Clin Cancer Res* **10**: 272-284

Lee MS, Hanspers K, Barker CS, Korn AP, McCune JM (2004) Gene expression profiles during human CD4+ T cell differentiation. *Int Immunol* **16**: 1109-1124

Manalo DJ, Rowan A, Lavoie T, Natarajan L, Kelly BD, Ye SQ, Garcia JG, Semenza GL (2005) Transcriptional regulation of vascular endothelial cell responses to hypoxia by HIF-1. *Blood* **105**: 659-669

Ren B, Cam H, Takahashi Y, Volkert T, Terragni J, Young RA, Dynlacht BD (2002) E2F integrates cell cycle progression with DNA repair, replication, and G(2)/M checkpoints. *Genes Dev* **16**: 245-256

Rhodes DR, Yu J, Shanker K, Deshpande N, Varambally R, Ghosh D, Barrette T, Pandey A, Chinnaiyan AM (2004) Large-scale meta-analysis of cancer microarray data identifies common transcriptional profiles of neoplastic transformation and progression. *Proc Natl Acad Sci USA* **101**: 9309-9314

Shepard JL, Amatruda JF, Stern HM, Subramanian A, Finkelstein D, Ziai J, Finley KR, Pfaff KL, Hersey C, Zhou Y, Barut B, Freedman M, Lee C, Spitsbergen J, Neuberg D, Weber G, Golub TR, Glickman JN, Kutok JL, Aster JC, Zon LI (2005) A zebrafish bmyb mutation causes genome instability and increased cancer susceptibility. *Proc Natl Acad Sci USA* **102**: 13194-13199

Su AI, Cooke MP, Ching KA, Hakak Y, Walker JR, Wiltshire T, Orth AP, Vega RG, Sapinoso LM, Moqrich A, Patapoutian A, Hampton GM, Schultz PG, Hogenesch JB (2002) Large-scale analysis of the human and mouse transcriptomes. *Proc Natl Acad Sci USA* **99**: 4465-4470

van't Veer LJ, Dai H, van de Vijver MJ, He YD, Hart AA, Mao M, Peterse HL, van der Kooy K, Marton MJ, Witteveen AT, Schreiber GJ, Kerkhoven RM, Roberts C, Linsley PS, Bernards R, Friend SH (2002) Gene expression profiling predicts clinical outcome of breast cancer. *Nature* **415**: 530-536

Wu Q, Kirschmeier P, Hockenberry T, Yang TY, Brassard DL, Wang L, McClanahan T, Black S, Rizzi G, Musco ML, Mirza A, Liu S (2002) Transcriptional regulation during p21WAF1/CIP1-induced apoptosis in human ovarian cancer cells. *J Biol Chem* **277**: 36329-36337

Yu D, Cozma D, Park A, Thomas-Tikhonenko A (2005) Functional validation of genes implicated in lymphomagenesis: an in vivo selection assay using a Myc-induced B-cell tumor. *Ann NY Acad Sci* **1059**: 145-159

Zhan F, Huang Y, Colla S, Stewart JP, Hanamura I, Gupta S, Epstein J, Yaccoby S, Sawyer J, Burington B, Anaissie E, Hollmig K, Pineda-Roman M, Tricot G, van Rhee F, Walker R, Zangari M, Crowley J, Barlogie B, Shaughnessy JD Jr (2006) The molecular classification of multiple myeloma. *Blood* **108**: 2020-2028

**Supplementary Table 1. Clinical, histological and mutational description of the 24 tumour specimens used in genome-wide expression analysis.**

^a^Age at diagnosis; ^b^Tumour greatest diameter; ^c^WHO classification of thyroid tumours (DeLellis *et al*., 2004); ^d^RET/PTC fusion gene was later detected using microarray data and confirmed by Fluorescent *In Situ* Hybridisation (FISH).

Abbreviations: F, female; M, Male; PDTC, poorly differentiated thyroid carcinoma; fvPTC, follicular variant of papillary thyroid carcinoma; cPTC, classic papillary thyroid carcinoma; FTC, follicular thyroid carcinoma; Mi, minimally invasive; Wi, widely invasive

**Supplementary Table 2. Main characteristics of differentially under-expressed genes in poorly differentiated tumours *versus* thyroid normal tissues.**

**Supplementary Table 2. (continued)**

**Supplementary Table 2. (continued)**

**Supplementary Table 2. (continued)**

^a^Assigned in *EntrezGene*; ^b^Information taken from Online Mendelian Inheritance in Man (OMIM) or from *EntrezGene*; *P* values for difference in mean expression between tumours and normal tissues were calculated using an unpaired *t*-test.

Abbreviations: LBFC, lower bound of fold change

**
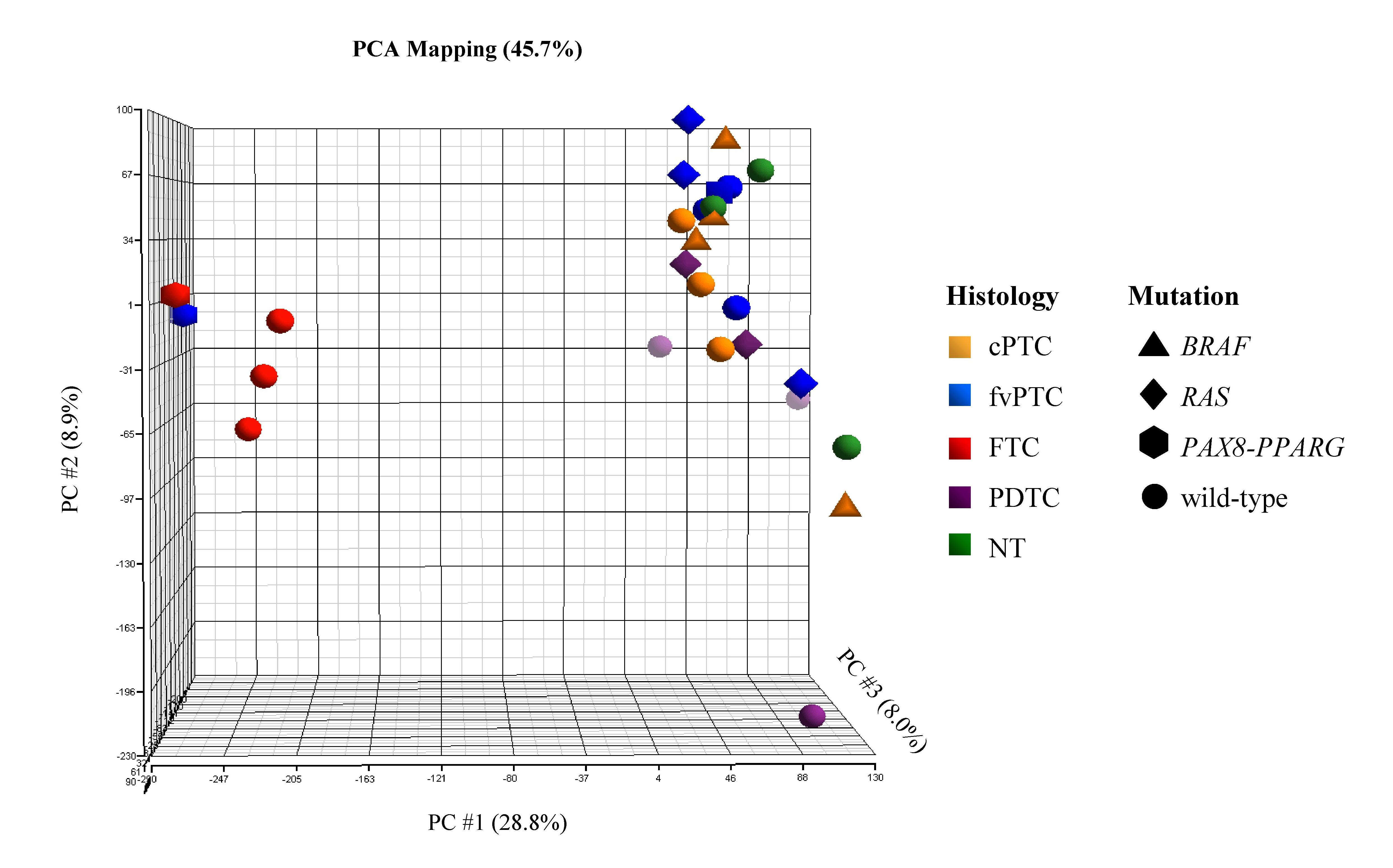
**

**Supplementary Figure 1. Three-dimensional representation of gene expression resemblance between samples using the Principal Components Analysis (PCA).** In this representation, variation in probe-sets data is represented by the three principal components (PC), thus the distance between samples indicates their gene expression similarity. Numbers in percentage indicate the degree of variability corresponding to each PC. Wild-type label denotes absence of mutations in screened genes.

Abbreviations: PDTC, poorly differentiated thyroid carcinoma; fvPTC, follicular variant of papillary thyroid carcinoma; cPTC, classic papillary thyroid carcinoma; FTC, follicular thyroid carcinoma; NT, normal thyroid tissues

**Supplementary Movie 1. Three-dimensional representation of gene expression resemblance between samples using the Principal Components Analysis (PCA).** Refer to supplementary Fig. 1.

**
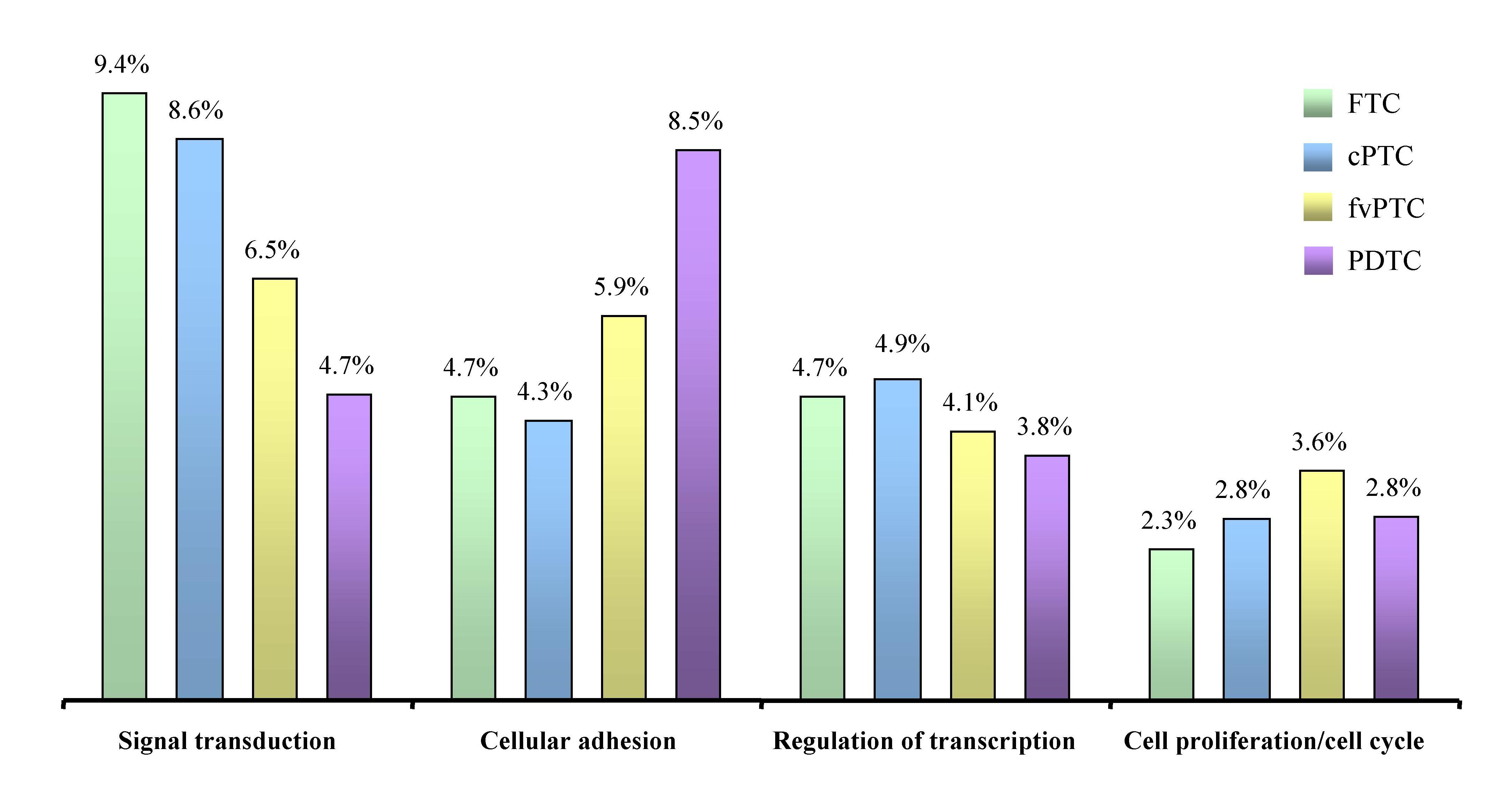
**

**Supplementary Figure 2. Representation of the most deregulated biological processes, between each thyroid tumour histotype and normal thyroid tissue.** Genes were classified in accordance with their biological role and the four processes represented by more differentially expressed genes, for each histotype, are indicated. Only one probe-set was considered for each gene.

Abbreviations: PDTC, poorly differentiated thyroid carcinoma; fvPTC, follicular variant of papillary thyroid carcinoma; cPTC, classic papillary thyroid carcinoma; FTC, follicular thyroid carcinoma
